# Insecticidal activity of monoamide compounds from *Humulus scandens* against *Spodoptera frugiperda*

**DOI:** 10.3389/fpls.2025.1573810

**Published:** 2025-09-18

**Authors:** Yuxuan Liu, Xiaoyun Wu, Fengchao Li, Deqiang Qin, Xi Gao, Guoxing Wu, Xiaoping Qin

**Affiliations:** ^1^ State Key Laboratory for Conservation and Utilization of Bio-Resources in Yunnan, College of Plant Protection, Yunnan Agricultural University, Kunming, China; ^2^ College of Water Conservancy, Yunnan Agricultural University, Kunming, China

**Keywords:** *Humulus scandens*, *Spodoptera frugiperda*, insecticidal activity, acetylcholinesterase, enzyme kinetics

## Abstract

*Spodoptera frugiperda*, a globally invasive pest, currently lacks effective control measures beyond certain chemical pesticides. Previous reports have demonstrated the effectiveness of *Humulus scandens* against various agricultural pests. Here, our aim was to derive toxic compounds and explore the insecticidal potential of the botanical plant *H. scandens* against *S. frugiperda*. Through activity-guided isolation, two monoamide compounds, *N*-*p*-coumaroyl tyramine **(1)** and *N*-trans-feruloyl tyramine **(2)**, were identified from the EtOAc extract of *H. scandens*. The results showed that both compounds **1** and **2** exhibited good insecticidal effects on *S. frugiperda* under both contact and dietary toxicity methods, with significant dose-dependent effects; however, compound **2** demonstrated stronger insecticidal activity. On the other hand, both compounds exhibited stronger contact toxicity than ingestion toxicity, with compound **2** having an LC_50_ (50% lethal concentration) of 47.97 μg/mL. Insecticidal mechanism studies revealed that both compounds act on the acetylcholinesterase (AChE) of *S. frugiperda*, with compound **2** showing stronger inhibition (50% inhibitory concentration, IC_50_ = 19.71 ± 1.98 μg/mL). Kinetic studies of the compounds on AChE indicated that both monoamides are reversible competitive inhibitors of AChE, with Ki values of 23.76 μg/mL and 20.79 μg/mL, respectively. This study revealed the active insecticidal compounds from *H. scandens* and their mechanisms of action. These findings provide new insights into the secondary metabolites of *H. scandens* which may serve as a basis for the development of novel plant based insecticides. Furthermore, these results suggested the use of these two bioactive compounds in the integrated management of *S. frugiperda* as alternatives to synthetic insecticides.

## 1 Introduction

*Spodoptera frugiperda*, commonly called fall armyworm originated from the tropical and subtropical regions of the USA, and is widely distributed across the continent. It is a significant trans-boundary migratory pest affecting food crops such as corn (Sparks, 1979; Todd and Poole, 1980). With the increasing frequency of international trade and the strong migratory and dispersive abilities of this pest (Chapman et al., 2017; Cock et al., 2017), *S. frugiperda* has invaded 46 countries or regions in Africa (CABI, 2016) and nine Asian countries, including China (National Agricultural Technology Extension Service Center, 2019a). Since its confirmation of invasion in Yunnan Province, China, in January 2019 (National Agricultural Technology Extension Service Center, 2019b), *S. frugiperda* had spread to 617 counties in 16 provinces by May 21, 2019, covering an area of 126,700 hectares and posing a significant threat to the production of corn in China.

Currently, the primary means of controlling *S. frugiperda* remain chemical methods (Fernandes et al., 2019), supplemented by comprehensive management through agricultural practices, biological control, and physicochemical induction (Yang et al., 2019). Commonly used pesticides include halofenozide, chlorantraniliprole, cis-permethrin, and carbosulfan (Lewter and Szalanski, 2007), while emamectin benzoate, acephate, spinosad, and fenpropathrin also showed good control effects on *S. frugiperda* (Zhao et al., 2019). Although chemical pesticides are highly where as effective against *S. frugiperda*, their hazardous effects on the environment cannot be overlooked. The excessive use of chemical pesticides possess great risks to human health, and increasing pest resistance poses enormous challenges to the safe and efficient production of crops. Therefore, plant-based pesticides that are low in toxicity, easily degradable, highly effective, and environmentally friendly have recently attracted attention of experts. The search for compounds with significant bioactive activities from natural plant products is a popular research direction in chemistry, pharmacology, and biological pesticides (Yang et al., 2022; Prastiwi et al., 2023; Saravanan, 2022). For example, azadirachtin which is isolated from *Azadirachta indica* is considered the most successful plant-based pesticide because of its excellent antifeedant activity (Xu et al., 2017). Additionally, plant-based insecticides such as celangulin, rotenone, and matrine, which have broad-spectrum insecticidal effects, are less prone to resistance, have low pesticide residues, and have also been widely used in agricultural production (Liu and Liu, 2021; Zhang et al., 2023; Fan et al., 2022; Wu et al., 2019). Therefore, it is highly practical to search for chemical components with insecticidal activity against *S. frugiperda* from natural products and to expand the means of controlling this pest.

*Humulus scandens* (Lour.) Merr., a herbaceous plant belonging to the genus Humulus in the Moraceae family, prefers shade and moisture and can adapt to various soil textures and climatic conditions. It has strong vitality and regeneration abilities and has been used as a traditional Chinese medicine for a long time in China (Hou, 1984). Studies have confirmed that *H. scandens* has good antibacterial, anti-inflammatory, anti-tubercular, antioxidant, and antitumor activities (Lewis et al., 1949; Kare, 1951; Ding et al., 2009; Gao et al., 2010). The secondary metabolites of *H. scandens* are complex and diverse. Predecessors have isolated or identified more than 170 compounds from various parts of the plant, with terpenoids and flavonoids being the predominant classes. Representative chemical components include lupulone, humulone, cosmosiin, and vitexin (Gao, 2015; Yang, 2015; Xu et al., 2014). The diversity of structures determines the diversity of functions. Therefore, we selected *H. scandens* as the plant material to explore its bioactive components or lead compounds with potential as plant-based pesticides.

Currently, there are few studies on the biological activity of *H. scandens* in the field of plant protection, and most related research has focused on allelopathic effects (Zhu et al., 2022). In the field of insecticides, only a limited number of studies have shown that extracts of *H. scandens* have certain biological activity against *Tetranychus cinnabarinus*, *Culex pipiens pallens larvae*, and *Aphis gossypii Glover* (Zhang et al., 2009; Huang et al., 2000; Sun et al., 2012), but the specific active components and mechanisms of action remain unclear. Therefore, in this study, we first investigated whether *H. scandens* has biological activity against *S. frugiperda*. After confirming the activity, we isolated and identified two monoamide compounds through activity-guided isolation and determined the active insecticidal compounds of *H. scandens*. Finally, we explored the toxic mechanisms of these two compounds against *S. frugiperda* in combination with symptomatic observations. This study provides a certain material basis and experimental evidence for developing *H. scandens* into a novel plant-derived insecticide.

## 2 Materials and methods

### 2.1 Instruments and reagents

The AV-500 MHz and 600 MHz nuclear magnetic resonance (NMR) spectrometers used in this experiment were purchased by Bruker, Switzerland. The 1290 UPLC/6540 Q-ToF liquid chromatography-mass spectrometry (LC-MS) system was from Agilent, USA. Preparative high-performance liquid chromatography (HPLC) was performed Hanbon Sci & Tech Co., Ltd., Jiangsu, China. The rotary evaporator was purchased by Heidolph, Germany. The vacuum diaphragm pump was from Vacuum, Germany. The low-temperature circulating cooling pump was manufactured by Eyela, Japan. The UV detector was produced by Anting Electronic Instrument Factory, Shanghai, China. The high-speed refrigerated centrifuge HC-3018R was manufactured by Zhongjia Scientific Instrument Co., Ltd., Anhui, China. The electronic balance FA2004 was from Jingke Instrument Co., Ltd., Shanghai, China. The microplate reader Varioskan LUX was from ThermoFisher.

Normal-phase silica gel (60-80, 200-300, 300–400 mesh) and thin-layer chromatography silica gel plates were manufactured by Qingdao Marine Chemical Co., Ltd., China. Reverse-phase silica gel RP-18 (40-63 μM) was from Merck, Germany. MCI filler (75-150 μM) was sourced from Chengdu Kexue Biochemical Co., Ltd., China. The gel Sephadex LH-20 (40-70 μM) was from Pharmacia, USA. The YMC-Pack ODS-A column (5 μm, 10×250 mm) was provided by Kemitech Co., Ltd., Shenzhen. Deuterated reagents were from Merck, USA. The acetylcholinesterase kit is provided by Shanghai Shenggong Biotech Co., Ltd. Acetylcholine was from Fluka Chemical Co., USA. 5,5’-Dithio-bis(2-nitrobenzoic acid) was from Biological Engineering Co., USA.

### 2.2 Plant material

The *H. scandens* used in this study were collected in July 2022 from Anyang, Henan Province, China (35°12′N, 113°37′E). The collected samples (No.2022017) were preliminarily identified by Dr. Guoxing Wu from Yunnan Agricultural University.

### 2.3 *S. frugiperda* collection and rearing

The *S. frugiperda* used in this study were reared artificially on corn leaves in a climate chamber by the Insect Toxicology Laboratory of Yunnan Agricultural University. Fourth-instar larvae were used for the experiments.

The *S. frugiperda* used in this study were artificially reared for multiple generations on corn leaves in a controlled environment chamber at the Insect Toxicology Laboratory of Yunnan Agricultural University (temperature: 26°C, relative humidity: 60%, light/dark = 14 h:10 h). Fourth-instar larvae were used for the experiments.

### 2.4 Extraction and isolation

After drying and pulverizing the *H. scandens* (20kg) collected from the wild, it was soaked in MeOH (30L) and extracted three times (24 h each time) at room temperature. All the extracts were combined and concentrated under reduced pressure to yield 2 kg of MeOH crude extract. The crude extract was then fully dissolved in water and extracted with equal volumes of PE, EtOAc, and n-BuOH (three times each) to obtain the crude extracts of the PE layer (253g), EtOAc layer (372g), and n-BuOH layer (324g). On the basis of activity test results of the crude extracts, an activity-guided approach was employed to further separate the EtOAc layer extract of *H. scandens*.

Using a silica gel column (10×100 cm), silica gel chromatography was performed on the EtOAc layer of *H. scandens*, which was eluted with a gradient of PE-EtOAc (50:1 to pure EtOAc). This resulted in 12 fractions (Lx.1-Lx.12).

The Lx.9 fraction (18.9 g) was first purified by MCI reverse-phase column chromatography (eluting with 10% MeOH-H_2_O to pure MeOH), yielding 9 sub fractions (Lx.9A-Lx.9I). The Lx.9G sub fraction (800 mg) was then subjected to normal-phase silica gel column chromatography (eluting with PE-EtOAc 15:1 to pure EtOAc), resulting in 4 sub fractions (Lx.9G1-Lx.9G4). The Lx.9G3 sub fraction (200 mg) was further purified by gel column chromatography (eluting with pure MeOH), and a relatively pure sample was obtained. This sample was then purified by HPLC (using 37% CH3CN as the organic phase, 0.1% FA to the aqueous phase.), ultimately yielding compound **1** (117 mg, tR = 15 min) and compound **2** (71 mg, tR = 37 min).

The structures of compounds **1** and **2** were determined by measuring their ^1^H, ^13^C, and DEPT, NMR spectral data, as well as mass spectral data.

### 2.5 Antifeedant activity test of extracts against *S. frugiperda*

The leaf disc method (Tang and Wang, 2007) was used to determine the antifeedant activity of crude extracts from the PE, EtOAc, and n-BuOH fractions of *H. scandens* against 4th instar larvae of *S. frugiperda*. The test fractions were prepared at a concentration of 10 mg/mL, with acetone serving as the solvent control. Leaf discs (d=1.2 cm) were soaked in the corresponding treatments for 3 s and allowed to dry before use. Once the larvae consumed more than 80% of the leaf area, the new treated leaf discs were replaced. Each treatment was repeated three times. The Petri dishes were placed in an incubator at 25°C to 30°C. After 8, 24, and 48 h, the area of the leaf consumed by each larva was measured via grid paper, and the antifeedant rate was calculated.


$$\begin{array}{c} \text{Antifeedant rate}\left(\%\right)\\ =\left(\text{Control leaf area consumed}-\text{Treated leaf area consumed}\right)/\\ \text{Control leaf area consumed}\times 100\% \end{array}$$

### 2.6 Insecticidal activity and toxicity evaluation of compounds against *S. frugiperda* through different modes of action

#### 2.6.1 Evaluation of the dietary toxicity of compounds against *S. frugiperda*

The dietary toxicity of the compounds against *S. frugiperda* was determined via the artificial feed film method (Lu, 2022). Take 1g of artificial feed and place it in a petri dish, then add 0.1 ml of the liquid medicine (acetone as the solvent and concentration of 200 μg/mL for all compounds) was added to form a uniform film on the surface of the feed. After allowing the feed to dry in a shaded area, one uniformly sized 4th instar *S. frugiperda* larva was introduced into each dish. Each treatment included 10 Petri dishes, and the experiment was repeated twice. Acetone was used as the solvent control, and a blank control was also used. Mortality was recorded at 24, 48, and 72 hours, and the corrected mortality rate was calculated.


$$\begin{array}{c} \text{Mortality}\left(\%\right)\\ =\left(\text{Mortality of treatment group}-\text{Mortality of control group}\right)/\\ \left(1-\text{Mortality of control group}\right)\times 100\% \end{array}$$

#### 2.6.2 Evaluation of the contact toxicity of compounds against *S. frugiperda*

The contact toxicity of the compounds against *S. frugiperda* was determined via the immersion method (Li et al., 2023). The compounds were prepared in solutions with a concentration of 200 μg/mL using acetone as the solvent. Uniformly sized 4th instar *S. frugiperda* larvae were selected and immersed in the solutions for 3–4 seconds. Each treatment included 10 Petri dishes, with one larva per dish, and the experiment was repeated three times. Acetone was used as the solvent control, and a blank control was also used. The number of dead insects was recorded at 24, 48, and 72 hours, and the corrected mortality rate was calculated.


$$\begin{array}{c} \text{Mortality}\left(\%\right)\\ =\left(\text{Mortality of treatment group}-\text{Mortality of control group}\right)/\\ \left(1-\text{Mortality of control group}\right)\times 100\% \end{array}$$

#### 2.6.3 Toxicity determination of compounds against the dietary toxicity of *S. frugiperda*

The compound concentrations used were 50, 100, 200, 400, and 800 μg/mL. The remaining procedures were the same as those described in section 2.6.1. After 72 hours, the number of dead insects was recorded. The experimental data were processed using Microsoft Excel and SPSS 20.0 software, with the inhibition rates converted into corresponding probability values. A toxicity regression equation was derived, and the LC_50_ value was calculated.

#### 2.6.4 Toxicity determination of contact activity of compounds against *S. frugiperda*

The compound concentrations were prepared as 50, 100, 200, 400, and 800 μg/mL, with the remaining procedures following those in section 2.6.2. The number of dead insects was recorded after 72 hours. The experimental data was processed using Microsoft Excel and SPSS 20.0 software, converting the inhibition rates into corresponding probability values to obtain the toxicity regression equation and calculate the LC_50_ value.

### 2.7 Observation of symptoms induced by bioactive compounds in *S. frugiperda*

Thirty 4th-instar *S. frugiperda* larvae of uniform size were selected and treated with a compound solution at the LC_90_ concentration using the immersion method. The control group was immersed in an equal amount of acetone. Symptoms were observed indoors with the naked eye and a dissecting microscope (5×), and photographs of typical symptoms were taken for recording (Xiao, 2014).

### 2.8 Determination of the acetylcholinesterase inhibitory activity of compounds in living *S. frugiperda*

The compounds were prepared in solutions at the LC_50_ and double the LC_50_, and 4th-instar *S. frugiperda* larvae were treated using the immersion method. Ten larvae were treated per concentration, with five replicates, and acetone was used as the solvent control. The Acetylcholinesterase (AchE) activity was measured following the operating procedures of the AchE assay kit (Shenggong Biotech Co., Shanghai, China.).

### 2.9 Determination of the IC_50_s of compounds against AchE from *S. frugiperda* (*in vitro*)

Pick approximately 0.1 g 4th-instar *S. frugiperda* larvae were placed in 1 mL extraction buffer, homogenized in an ice bath for 5 minutes, and then centrifuged at 10,000 rpm at 4°C for 10 minutes. The supernatant was collected and placed in an ice bath as the enzyme solution for testing.

The inhibitory activity of the compounds against AchE activity was determined, with chlorpyrifos serving as the positive control (Li et al., 2023). The compounds and chlorpyrifos (positive control) were dissolved in acetone and prepared into five concentration gradients for activity determination. The test compound solutions (4 µL) were mixed with the enzyme solutions (96 µL) (final compound concentrations of 3.75, 7.5, 15, 30, and 60 µg/mL), and incubated in a 96-well plate at 37°C for 2 hours. Subsequently, 1.5 mM acetylcholine (ACh) (50 µL) was added, and the mixture was further incubated at 37°C for 5 minutes. Finally, 0.3 mM 5,5’-dithiobis(2-nitrobenzoic acid) (DTNB) (50 µL) was added to terminate the reaction. The residual activity of AChE was then measured using a microplate reader at 412 nm.


$$\begin{array}{c} \text{Inhibition rate}\left(\%\right)\\ =\left(\text{OD control}-\text{OD treated}\right)/\text{OD control}\times 100\% \end{array}$$

### 2.10 Kinetic study of compound inhibition on AchE from *S. frugiperda*

Following the method of Li et al. (2020), four concentration gradients of enzyme solutions (0.125, 0.25, 0.5, 1.0 U/mL) were prepared in this study. After adding the compounds (at final concentrations of 3.75, 7.5, 15, 30, 60 µg/mL), the mixed enzyme solutions (100 µL each) were incubated in a 96-well plate at 37°C for 1 hour (with acetone as the solvent control). Subsequently, 1.5 mM acetylcholine (ACh) (50 µL) and 0.3 mM DTNB (50 µL) were added, and the time was immediately recorded. The OD values were accurately measured after 60 seconds at 412 nm using a microplate reader, and the reaction rate (ΔA/min) was calculated based on the change in OD value per minute. By plotting the relationship between the reaction rates of the compounds at different concentrations and the enzyme concentrations, and comparing the reaction velocities (v) with the relationship between different enzyme concentrations under different compound concentrations, the reversibility of the compound’s inhibition on AchE from *S. frugiperda* was assessed (Xiong et al., 2016).

Keeping the enzyme concentration constant, mixed enzyme solutions (100 µL each) with added compounds (at final concentrations of 3.75, 7.5, 15, 30, 60 µg/mL) were incubated in a 96-well plate at 37°C for 1 hour (with acetone as the solvent control). Subsequently, 0.3 mM DTNB (50 µL) and ACh at different concentrations (0.325, 0.75, 1.5, 3, 6 mM) were added, and the time was immediately recorded. The OD values were accurately measured after 60 seconds at 412 nm using a microplate reader, and the reaction rate (ΔA/min) was calculated based on the change in OD value per minute. Lineweaver-Burk double reciprocal plots were constructed to infer the inhibition type (Guo et al., 2018). The inhibition constant (Ki) of the compound was calculated by plotting the Dixon plot.

**Figure:**
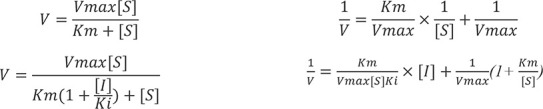


### 2.11 Data analysis

The experimental data were processed and statistically analyzed using Microsoft Excel and SPSS 20.0 software. Graphs were plotted using GraphPad Prism 8 software. Duncan’s multiple range test was used to compare the significance of differences, with p< 0.05 indicating significant differences.

## 3 Results and discussion

### 3.1 Study on the antifeedant activity of *H. scandens* crude extracts against *S. frugiperda*

**Figure 1:**
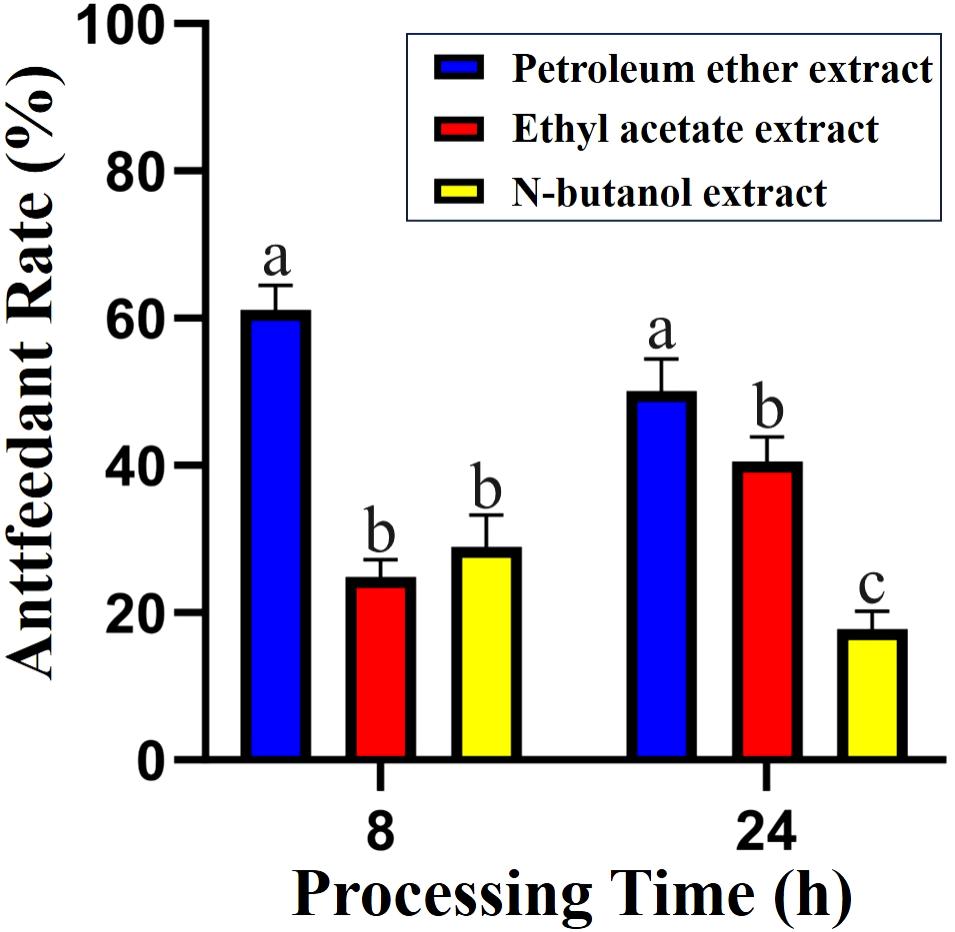
Antifeedant activity of *H. scandens* crude extracts against *S. frugiperda*.

To explore the biological activity of secondary metabolites from *H. scandens* against *S. frugiperda*, we investigated the biological activity of different fractions of *H. scandens* via the leaf disc method. The experimental results are shown in **Figure 1**. The results indicated that the PE, EtOAc, and n-BuOH crude extracts of *H. scandens* all had certain antifeedant effects on the 4th-instar larvae of *S. frugiperda*, with the overall effect being PE layer > EtOAc layer > n-BuOH layer, and there were significant differences among the three.

During the early treatment period (0 h-8 h), the antifeedant rate of the PE layer against *S. frugiperda* was 61.14%, which was significantly greater than that of the other two layers. However, over time passed, it decreased to 48.56% from 8h to 24h. Conversely, the results for the EtOAc layer were the opposite. The antifeedant rate of the EtOAc layer against *S. frugiperda* was only 24.91% during the 0h-8h period, but it increased by 16.65% from 8h to 24h. According to the research of Wang Qisen (Wang, 2016), the chemical components in PE extracts typically have low polarity and are volatile. On the basis of these findings, we speculate that during the initial treatment period, the PE layer crude extract, containing a large number of volatile components, strongly stimulated the olfactory or gustatory receptors of *S. frugiperda*, leading to antifeedant behavior. Over time, the low-polarity compounds continuously volatilized, resulting in a decrease in the antifeedant activity of the *H. scandens* PE layer. Sun et al. (2012) reported that *H. scandens* EtOAc extracts had certain contact toxicity against Lepidopteran pests such as *Plutella xylostella* at higher concentrations, and this effect was dose-dependent. So, we speculate that the active insecticidal components in *H. scandens* are concentrated mainly in the EtOAc layer. However, due to the low treatment concentration and complex composition of the crude extract, the effective components accounted for a relatively low proportion, resulting in the test insects only exhibiting significant antifeedant behavior without mortality after ingesting the EtOAc layer crude extract.

To further corroborate the insecticidal activity of the EtOAc fraction of *H. scandens* against *S. frugiperda*, we employed the artificial diet film method to determine the insecticidal activity of each fraction (L1-L12) obtained after crude fractionation of *H. scandens*, with the results presented in **Table 1**. At a concentration of 2 mg/mL, all fractions of the EtOAc fraction of *H. scandens*, except for Lx.1, exhibited certain insecticidal activity against *S. frugiperda*. Among them, Lx.9 demonstrated the highest insecticidal activity, reaching 53.76%, while the insecticidal rates of other fractions did not exceed 40%. Therefore, we conducted further separation of Lx.9 in an attempt to identify the active ingredient in *H. scandens* responsible for its insecticidal effect against *S. frugiperda*.

**Table 1:** The insecticidal activity of EtOAc fractions from *H. scandens* against *S. frugiperda*.

Fractions	Mortality (%)
1	–
2	17.57^g^
3	10.37^h^
4	22.54^f^
5	32.11^d^
6	29.68^e^
7	36.28^c^
8	41.73^b^
9	53.76^a^
10	39.25^b^
11	27.14.^e^
12	15.93^g^

Different letters indicate significant differences (P<0.05).

### 3.2 Structural identification of compounds

**Figure 2:**
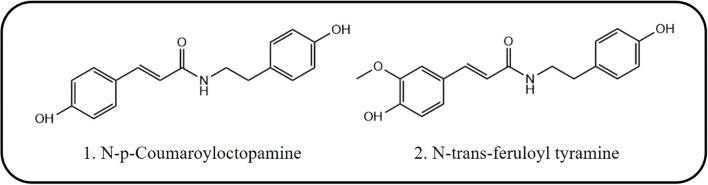
Chemical structures of the two compounds.

Compound **1**: *N*-*p*-coumaroyl tyramine, a white non-crystalline powder. ESI-MS m/z: 283.3624 [M+]. The NMR spectral data are as follows: ^1^H-NMR (500 MHz, MeOD) δH: 7.43 (d, *J* = 15.8 Hz, 1H), 7.43 (1H, d, *J*=15.8 Hz, H-7’), 7.37 (2H, d, *J*=8.8 Hz, H-2’, 6’), 7.02 (2H, d, *J*=8.4 Hz, H-2, 6), 6.78 (2H, d, *J*=8.8 Hz, H-3’, 5’), 6.70 (2H, d, *J*=8.4 Hz, H-3, 5), 6.37 (1H, d, *J*=15.7 Hz, H-8’), 3.45 (2H, t, *J*=6.7 Hz, H-7), 2.74 (2H, t, *J*=7.4 Hz, H-8), ^13^C-NMR (500 MHz, MeOD) δ: 169.24 (C-9), 160.52 (C-4), 156.92 (C-4’), 141.78 (C-7), 131.30 (C-1’), 130.73 (C-2’, 6’), 130.55 (C-2, 6), 127.71 (C-1), 118.41 (C-8), 116.71 (C-3’, 5’), 116.25 (C-3, 5), 42.55 (C-8’), 35.81 (C-7’). The data are consistent with the reference (Kim and Lee, 2003), confirming the structure of compound **1** (**Figure 2**).

Compound **2**: *N*-trans-feruloyl tyramine, a white non-crystalline powder.ESI-MS *m/z*: 314.1387 [M+H]^+^;^1^H-NMR (500 MHz, MeOD) δH: 7.42 (1H, d, *J* = 15.7 Hz, H-7’), 7.09 (1H, d, *J* = 1.9 Hz, H-2’), 7.04 (m, 2H, H-2,6), 7.00 (1H, dd, *J* = 8.2, 2.0 Hz, H-6’), 6.78 (1H, d, *J* = 8.1 Hz, H-5’), 6.71 (2H, m, H-3,5), 6.39 (1H, d, *J* = 15.7 Hz, H-8’), 3.86 (3H, S, H-OCH_3_), 3.45 (2H, t, *J* = 6.7 Hz, H-8), 2.74 (2H, t, *J* = 7.4 Hz, H-7), ^13^C-NMR (500 MHz, MeOD) δ: 169.17 (C-9’), 156.92 (C-4), 149.82 (C-4’), 149.27 (C-3’), 142.04 (C-7’), 131.29 (C-1), 130.74 (C-2,6), 128.26 (C-1’), 123.22 (C-6’), 118.73 (C-8’), 116.46 (C-5’), 116.26 (C-5), 111.51 (C-2’), 55.36 (-OCH_3_), 42.55 (C-8), 35.80 (C-7). The data are consistent with the previous study (Jiang and Ying, 2017), confirming the structure of compound **2** (**Figure 2**).

### 3.3 Determination of insecticidal activity and toxicity of compounds against *S. frugiperda* through different modes of action

**Figure 3:**
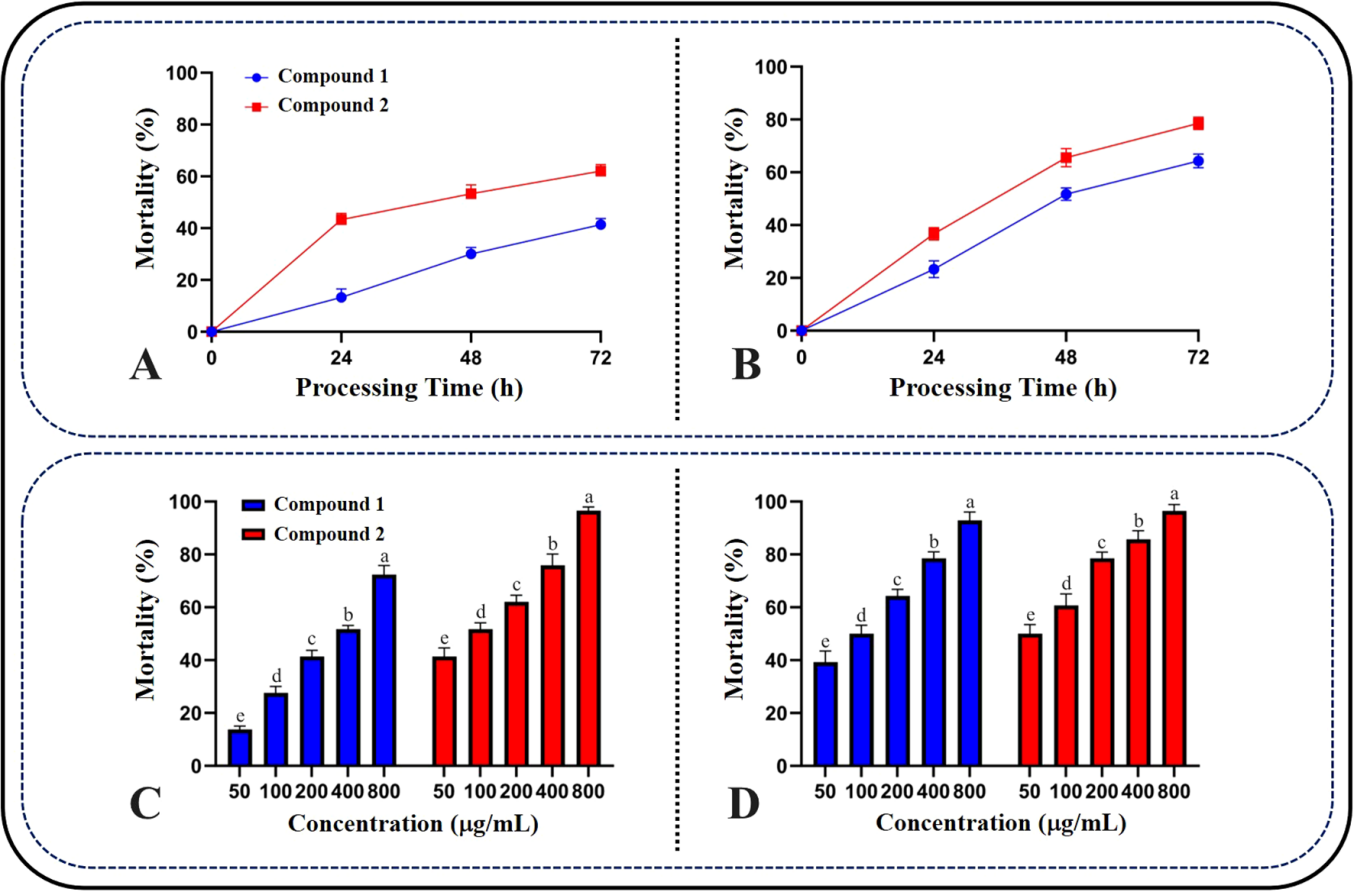
Insecticidal activity and toxicity evaluation of compounds against *S. frugiperda* in different modes of action. **(A)** Insecticidal activity of compound stomach poison against *S. frugiperda*. **(B)** Insecticidal activity of compounds contact against *S. frugiperda*. **(C)** Determination of Toxicity of Compounds Against *S. frugiperda* by stomach toxicity. **(D)** Determination of Toxicity of Compounds Against *S. frugiperda* by contact-killingt.

To verify the insecticidal activity of the two compounds against *S. frugiperda* and identify the optimal mode of action, we measured their dietary poisoning and contact toxicity against the pest at a concentration of 200 µg/mL. The results are shown in **Figures 3A, B**. Both compounds exhibited good insecticidal activity against *S. frugiperda* at this concentration, with the contact toxicity being significantly stronger than the dietary toxicity. Additionally, compared with compound **1**, compound **2** demonstrated significantly superior insecticidal activity.

To determine the specific insecticidal activities of the two compounds in this study, we conducted toxicity evaluations for both dietary poisoning and contact toxicity, with the results shown in **Figures 3C, D**. Both compounds exhibited a typical dose-dependent effect on the toxicity against *S. frugiperda*. Consistent with the initial screening results of insecticidal activity, both compounds demonstrated stronger contact toxicity than dietary poisoning did. Additionally, compound **2** showed more potent insecticidal activity than did compound **1**, with an LC_50_ of only 47.97 µg/mL for contact toxicity. The specific LC_50_ values for both compounds against *S. frugiperda* under different modes of action are presented in **Table 2**.

**Table 2:** Toxicity of the compounds to *S. frugiperda* in two modes of action.

	Compounds	Virulence regression equation	LC_50_ (μg/mL)	R^2^
Gastrotoxic activity	1	y = 46.962x - 66.683	305.25	0.9888
	2	y = 44.673x - 37.275	89.88	0.975
Poisoning activity	1	y = 45.083x - 38.738	92.96	0.9972
	2	y = 39.153x - 15.807	47.97	0.9829

From a structural perspective, the additional methoxy group at C-3’ in compound **2** maybe the active group. However, the specific mechanism underlying this enhanced insecticidal activity remains to be elucidated. In fact, many amide compounds exhibit good insecticidal activity against insects. For example, chlorantraniliprole, discovered by DuPont, possesses excellent insecticidal activity, along with advantages such as broad-spectrum effectiveness, low toxicity, and environmental friendliness, making it highly effective against Lepidopteran pests (Liu et al., 2023). Additionally, most carbamate pesticides feature a typical amide structure, such as carbofuran and methomyl. However, many of these pesticides have been banned due to their high toxicity. Therefore, identification of natural amide compounds isolated from *H. scandens* may provide new insights into the development of amide insecticides.

### 3.4 Observation of the toxicity symptoms induced by the compounds

Insecticide symptomatology is the science of studying the different toxic symptoms caused by insecticides in target insects, and it is the initial and most important step in elucidating the mechanisms of insecticide action. Different mechanisms of action are the main factors determining different symptoms, and the toxicological effects of insecticidal components can be inferred based on the symptoms of poisoning (Xiao, 2014).

**Figure 4:**
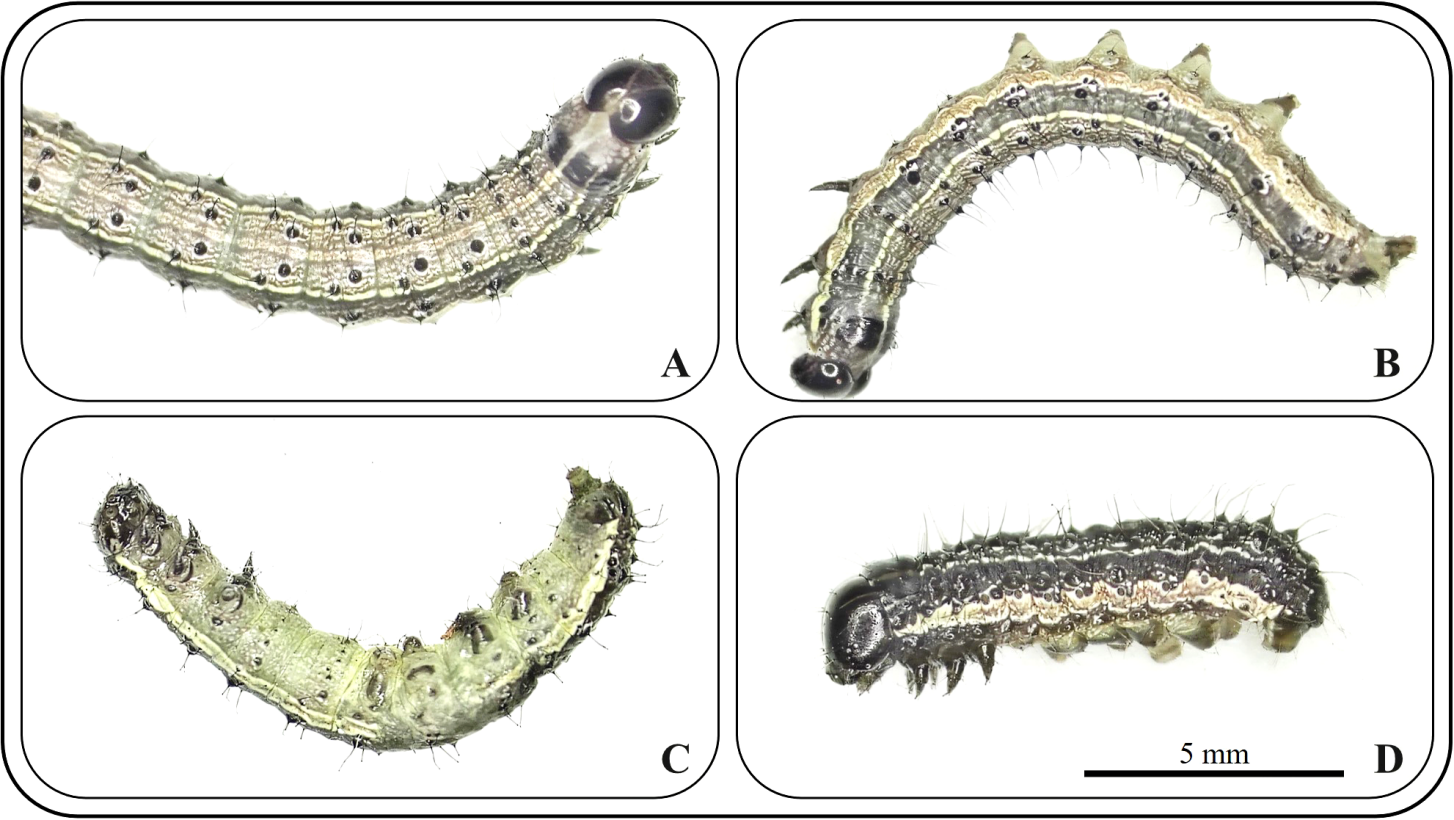
Symptoms of *S. frugiperda* poisoning at different stages. **(A)** excitement; **(B)** spasm; **(C)** paralysis; **(D)** death.

To preliminarily determine the insecticidal mechanisms of the two compounds against *S. frugiperda* in this study, we observed the symptomatology of poisoned insects treated with the compounds, and the results are shown in **Figure 4**. In the early stages of compound exposure, the test insects continuously twisted their bodies and lifted their heads high, resulting in a state of high excitement (**Figure 4A**). They subsequently gradually became limp, lay on their sides in the Petri dish, and exhibited typical convulsive behavior (**Figure 4B**). They then gradually became anesthetized and unconscious, with some insects showing slight tremors when lightly touched with a dissecting needle. When they turned onto their backs, they were unable to right themselves, and under a dissecting microscope, slight tremors could be observed in the tarsi, mouthparts, or cerci (**Figure 4C**). Finally, they were anesthetized to death, and no response was observed regardless of how they were prodded with a dissecting needle, with the insect bodies becoming noticeably wrinkled and shortened (**Figure 4D**).

Carbamate insecticides are typical AchE inhibitors. According to the research by Ma (2002), the toxic symptoms of carbamate insecticides can be clearly divided into four stages: excitement, convulsion, coma, and death. The symptoms observed in this study for the two compounds are highly similar to those of carbamate pesticide poisoning. Therefore, we initially hypothesized that compounds **1** and **2** cause death by inhibiting the AchE activity of the test insects.

### 3.5 Inhibitory activity and IC_50_ determination of compounds on AchE of *S. frugiperda*

**Figure 5:**
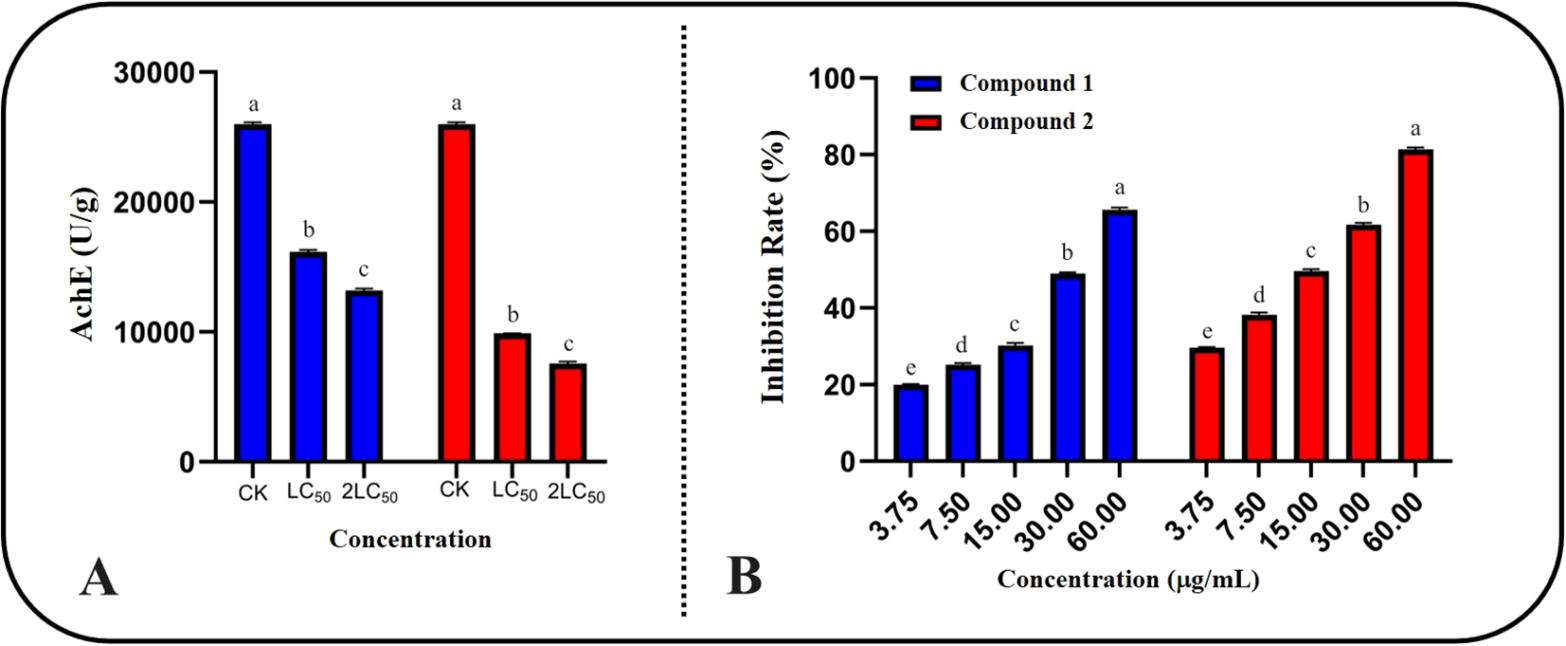
Inhibitory activity of compounds on AchE of *S. frugiperda*. **(A)** The inhibitory activity of compound LC50 and 2LC50 on AchE of *S. frugiperda*. **(B)** The inhibitory activity of compounds on AchE *in vitro* conditions.

To verify the conclusions drawn from our study on the symptomatology of compound poisoning, we measured the AchE activity of *S. frugiperda* treated with the compounds, and the results are shown in **Figure 5A**. After treatment with compounds **1** and 2 at LC_50_ and double the LC_50_, the AchE activity of the test insects significantly decreased, further suggesting that the insecticidal target of these two amide compounds in *S. frugiperda* is AchE. The AchE activity after treatment with compound **2** was significantly lower than that after treatment with compound **1**, which is consistent with the previous insecticidal activity results.

To further characterize the specific inhibitory effects of the two compounds on AchE, we determined their IC_50_ values under *in vitro* conditions, and the results are shown in **Figure 5B**. Both compounds clearly inhibited AchE in *S. frugiperda* in a dose-dependent manner. At the same concentration, compound **2** had stronger inhibitory effects on AchE than compound **1**. The specific IC_50_ values are presented in **Table 3**. The IC_50_ values for the two compounds were 23.34 ± 2.72 μg/mL and 19.71 ± 1.98 μg/mL, respectively, while the IC_50_ for chlorpyrifos was 2.57 ± 0.37 μg/mL. Although compounds **1** and **2** both exhibited good inhibitory effects on AchE, there is still a gap between them and the development of finished pesticides. They can be studied as lead compounds for the creation of new pesticides.

**Table 3:** Toxicity of the compounds to *S. frugiperda* in two modes of action.

Compounds	IC_50_ (μg/mL)	R^2^
1	23.34 ± 2.72^c^	0.9834
2	19.71 ± 1.98^b^	0.9692
chlorpyrifos	2.57 ± 0.37^a^	0.9763

Different letters indicate significant differences (P<0.05).

### 3.6 Kinetics study of compounds on AchE of *S. frugiperda*

**Figure 6:**
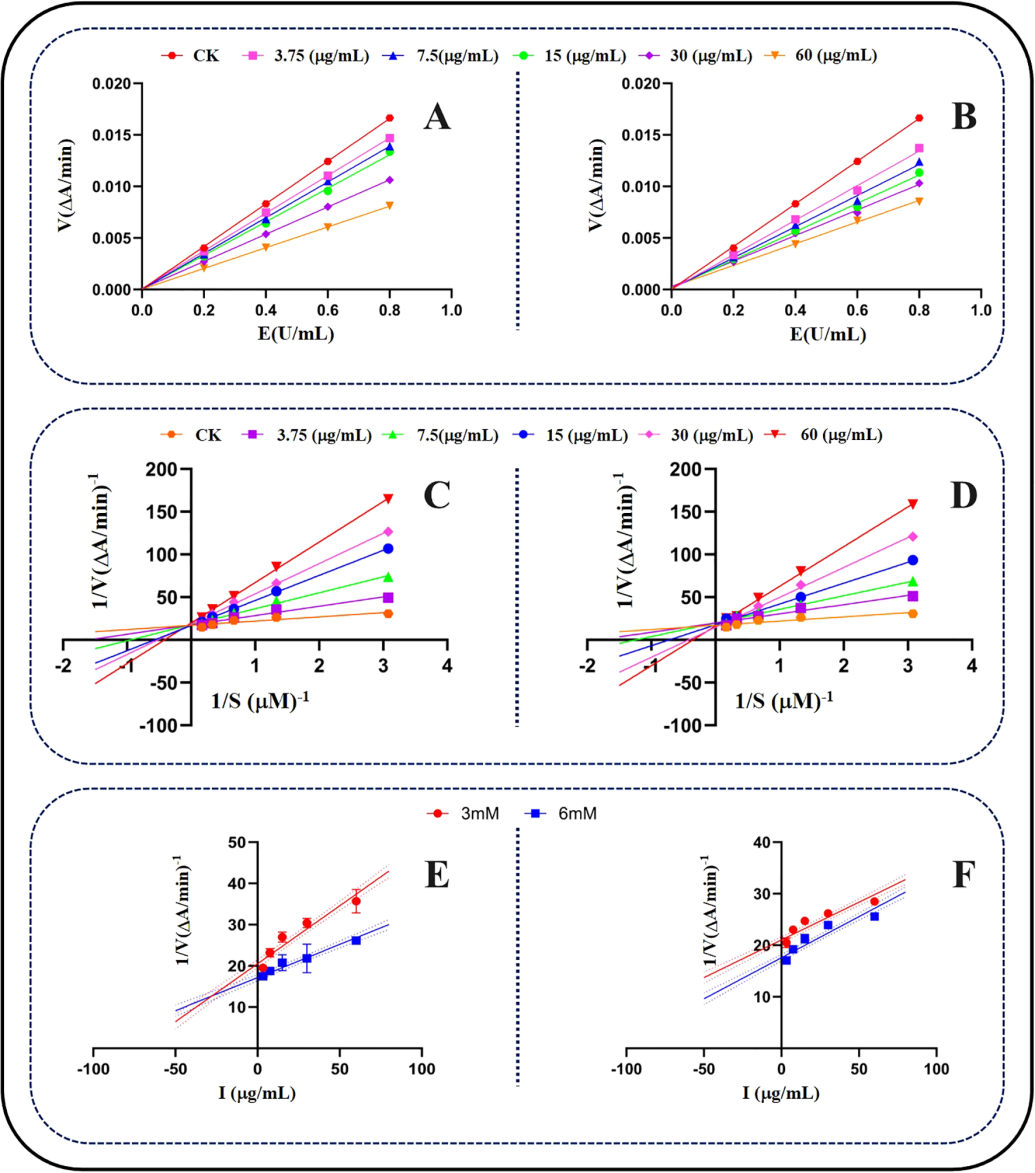
Kinetic study of the effects of compounds on AchE of *S. frugiperda.* **(A, B)** The inhibition type of compounds on AchE **(C, D)** Double Reciprocal Curve of AchE Inhibitory Effect of Compounds **(E, F)** The Ki of compounds on AchE.

After identifying the insecticidal target of compounds **1** and **2** as the AchE of *S. frugiperda*, we further explored the inhibitory mechanism of the compounds on AchE by studying their enzyme kinetics, the results are shown in **Figure 6**. By measuring the enzymatic reaction rates of AchE treated with different concentrations of compounds at various enzyme concentrations (**Figures 6A, B**), we found that the relationship between the enzymatic reaction rates of compounds **1** and **2** at different concentrations and enzyme concentrations was a straight line, and the lines for different concentrations of both compounds intersected at the origin, with the slope decreasing as the compound concentration increased. The kinetic results for both compounds showed the same trend. These results indicate that both compounds **1** and **2** are reversible inhibitors of AchE (Li et al., 2023).

After confirming that both compounds are reversible inhibitors of AchE, we analyzed the relationship between the enzymatic reaction rates of AchE treated with different concentrations of compounds and the substrate (Ach) via Lineweaver-Burk double reciprocal plots to explore their inhibition modes. According to the Lineweaver-Burk plots in **Figures 6C, D**, the lines fitted at different concentrations of compounds **1** and **2** also showed the same trend, with a unique slope, and the lines for different concentrations of both compounds intersected on the Y-axis. This indicates that as the concentration of compounds **1** and **2** increased, the fitted curves had the same intercept on the Y-axis (1/Vmax), but the intercepts on the X-axis (-1/Km) increased. This suggests that as the compound concentration increased, the Michaelis constant (Km) of AchE increased, whereas the Vmax remained unchanged. Therefore, we can conclude that both compounds **1** and **2** are competitive inhibitors of AchE.

After confirming that both compounds are competitive inhibitors of AchE, we determined their inhibition constants (Ki). The inhibition constant directly reflects the affinity between the enzyme inhibitor and its host enzyme, with a lower Ki value indicating a greater affinity of the inhibitor for the enzyme. Based on the slopes of the plots of the reciprocal of the maximum initial velocity of the enzymatic reaction versus different compound concentrations in the Dixon plots (**Figures 6E, F**), the Ki value for compound **1** was 23.76 μg/mL, and the Ki value for compound **2** was 20.79 μg/mL. These results are consistent with previous findings on the inhibitory activity of these two compounds against AchE. Thus, the reason why compound **2** exhibits stronger insecticidal activity against *S. frugiperda* than does compound **1** has been revealed. This is because the additional methoxy group at C-6 in compound **2** enhances its affinity for AchE, resulting in a stronger inhibitory effect on AchE and, consequently, stronger insecticidal activity.

## 4 Conclusion

In this study, two amides were isolated from *H. scandens*, and their insecticidal activity and mechanism against *S. frugiperda* were investigated. The experimental results demonstrated that both monoamide compounds exhibited good contact toxicity against *S. frugiperda*, with compound **2** showing stronger insecticidal activity than compound **1**, with an LC_50_ of only 47.97 μg/mL in contact toxicity tests. Furthermore, symptomatological studies and *in vivo* enzyme activity experiments indicated that both compounds **1** and **2** targeted the AchE of *S. frugiperda*. Specifically, compound **2** had an IC_50_ of 19.71 ± 1.98 μg/mL against AchE. Additionally, kinetic studies of the compounds on AchE revealed that both monoamide compounds were reversible and competitive inhibitors of AchE, with Ki values of 23.76 and 20.79 μg/mL, respectively. In summary, this study elucidated the insecticidal active components and their mechanisms of action from *H. scandens* against the invasive pest *S. frugiperda*, further enriching the secondary metabolites of *H. scandens* and enhancing its medicinal value in the field of plant protection. This study provides new insights for better control of the global invasive pest *S. frugiperda*, and holds great significance for the research and development of new plant-derived pesticides.

## Data Availability

The original contributions presented in the study are included in the article/**Supplementary Material**. Further inquiries can be directed to the corresponding authors.
