# Supplementary material for: Insecticidal activity of monoamide compounds from Humulus scandens against Spodoptera frugiperda
Source: Front Plant Sci. 2025 Sep 18;16:1573810. doi: 10.3389/fpls.2025.1573810 (PMC12488629; doi:10.3389/fpls.2025.1573810)
Supplement: Supplementary file 1 [file DataSheet1.pdf]

# *Supplementary Material*

**Figure S1** $^{1}$H NMR spectrum of compound **1** in MeOD

**Figure S2** $^{13}$C NMR (DEPT 135 and 90) spectrum of compound **1** in MeOD

**Figure S3** $^{1}$H NMR spectrum of compound **2** in MeOD

**Figure S4** $^{13}$C NMR (DEPT 135 and 90) spectrum of compound **2** in MeOD

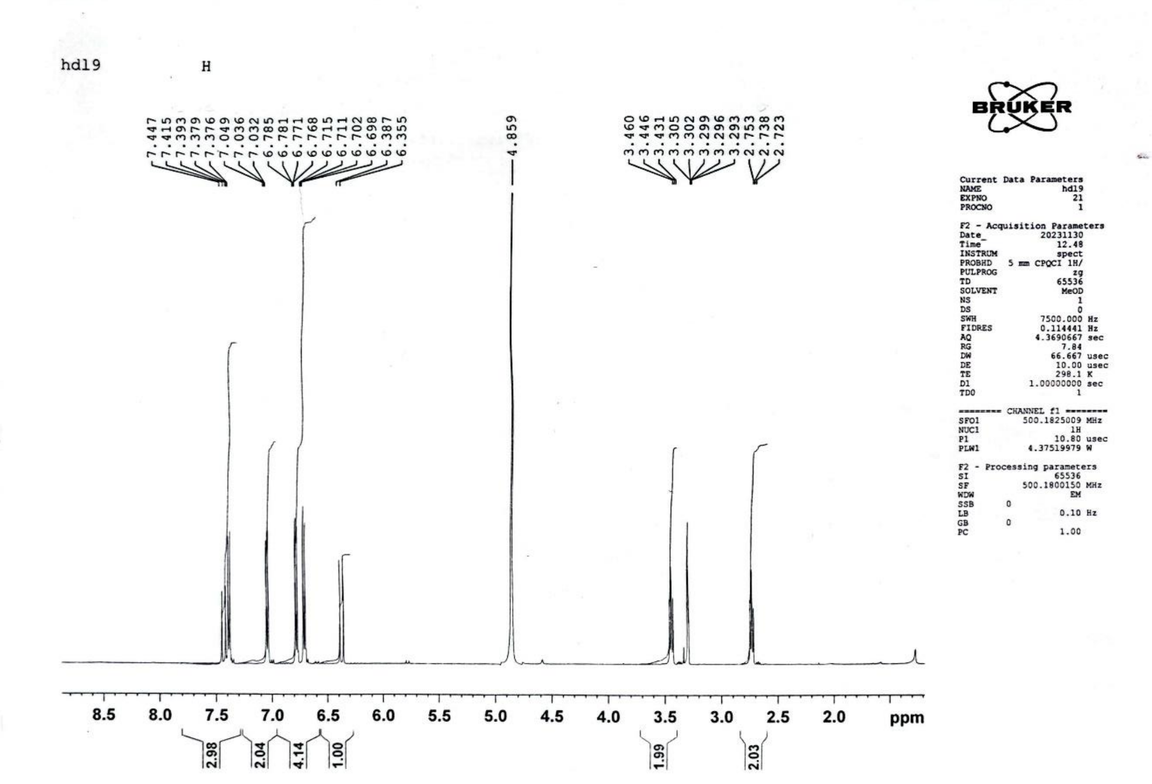

**Figure S1** $^{1}$H NMR spectrum of compound **1** in MeOD

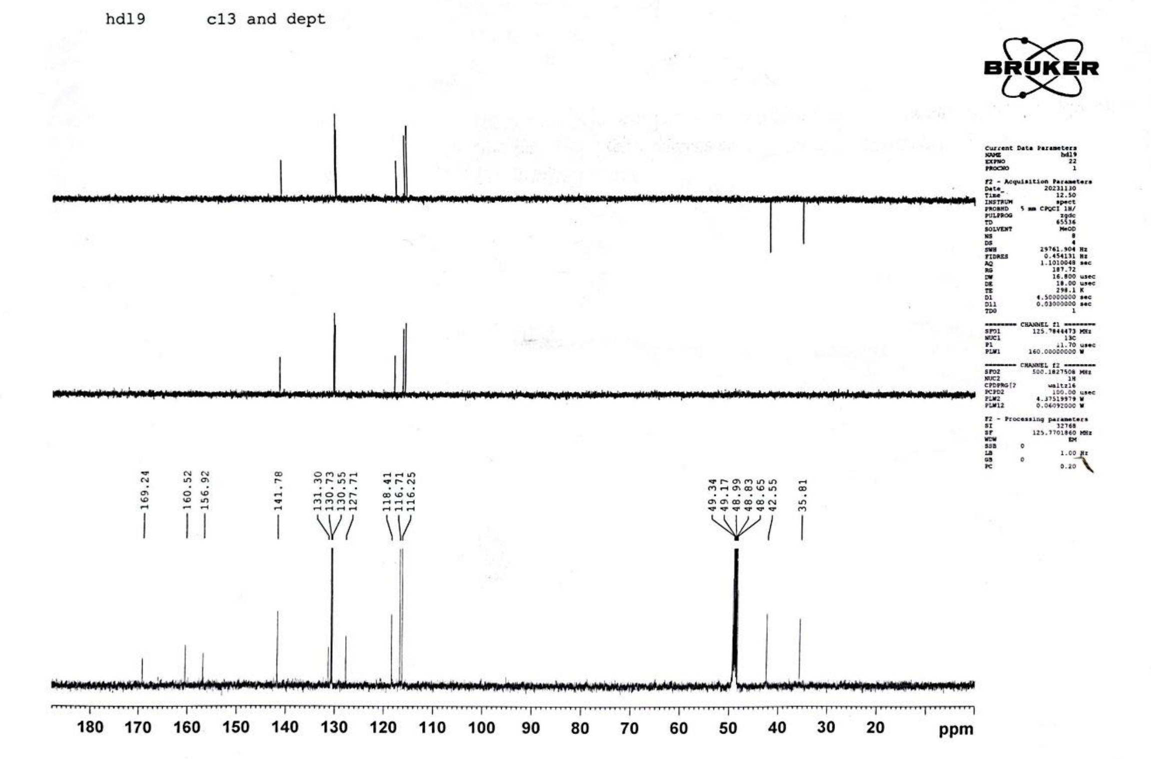

**Figure S2** $^{13}$C NMR (DEPT 135 and 90) spectrum of compound **1** in MeOD

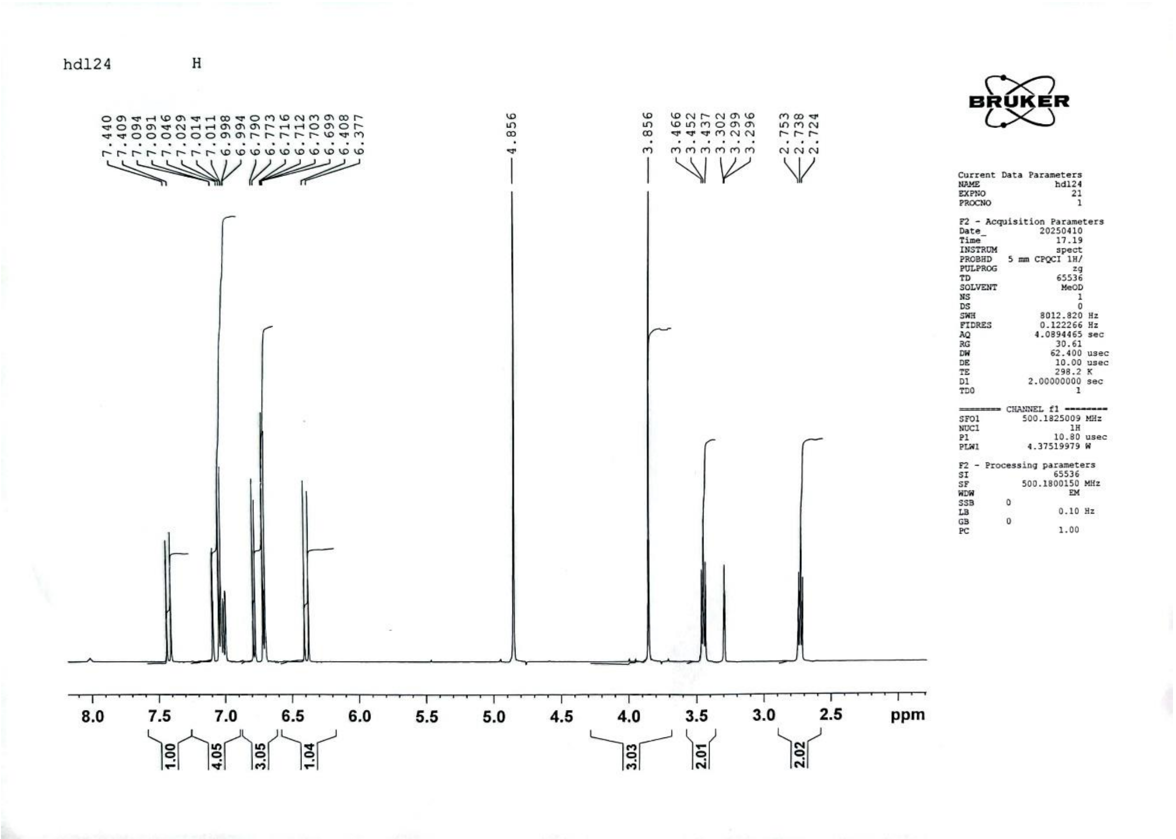


**Figure S3** $^{1}$H NMR spectrum of compound **2** in MeOD

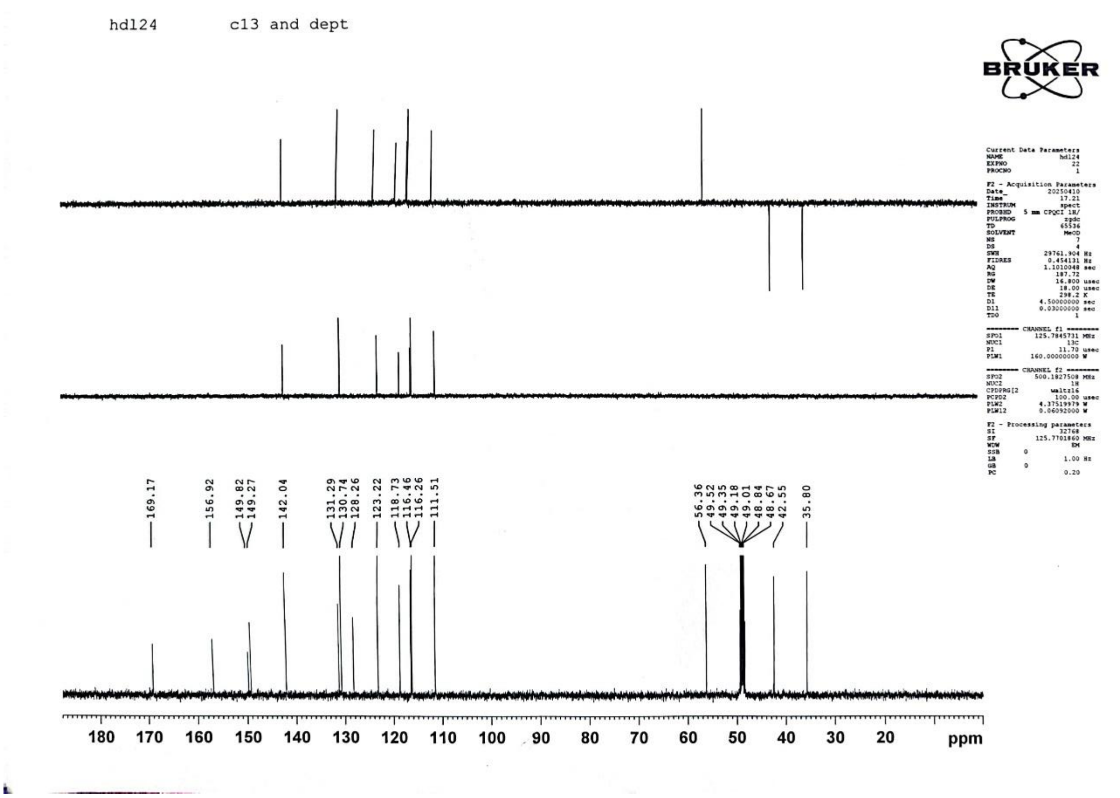


**Figure S4** $^{13}$C NMR (DEPT 135 and 90) spectrum of compound **2** in MeOD
